# Khat Chewing and Restrictive Dietary Behaviors Are Associated with Anemia among Pregnant Women in High Prevalence Rural Communities in Eastern Ethiopia

**DOI:** 10.1371/journal.pone.0078601

**Published:** 2013-11-04

**Authors:** Haji Kedir, Yemane Berhane, Alemayehu Worku

**Affiliations:** 1 College of Health and Medical Science, Haramaya University, Harar, Ethiopia; 2 Addis Continental Institute of Public Health, Addis Ababa, Ethiopia; 3 School of Public Health, Addis Ababa University, Addis Ababa, Ethiopia; Université Catholique de Louvain, Belgium

## Abstract

**Background:**

Anemia affects a high proportion of pregnant women in the developing countries. Factors associated with it vary in context. This study aimed to determine the prevalence and predictors of anemia among pregnant women in the rural eastern Ethiopia.

**Methods:**

A community-based cross-sectional study was done on 1678 pregnant women who were selected by a cluster random sampling technique. A pregnant woman was identified as anemic if her hemoglobin concentration was <11 g/dl. Data were collected in a community-based setting. Multilevel mixed effect logistic regression was used to determine the adjusted odds ratios (AOR) with 95% confidence intervals (CI) for the predictors of anemia.

**Results:**

Anemia was observed among 737(43.9%) of the 1678 pregnant women studied (95% CI 41.5%–46.3%). After controlling for the confounders, the risk of anemia was 29% higher in the women who chewed khat daily than those who sometimes or never did so (AOR, 1.29; 95% CI, 1.02–1.62). The study subjects with restrictive dietary behavior (reduced either meal size or frequency) had a 39% higher risk of anemia compared to those without restrictive dietary behavior (AOR, 1.39; 95% CI, 1.02–1.88). The risk of anemia was increased by 68% (AOR, 1.68; 95% CI, 1.15–2.47), and 60% (AOR, 1.60; 95% CI, 1.08–2.37) in parity levels of 2 births and 3 births, respectively. Compared to the first trimester, the risk of anemia was higher by two-fold (AOR, 2.09; 95% CI, 1.46–3.00) in the second trimester and by four-fold (AOR, 4.23; 95% CI, 2.97–6.02) in the third trimester.

**Conclusion:**

In this study, two out of five women were anemic. Chewing khat and restrictive dietary habits that are associated with anemia in the setting should be addressed through public education programs. Interventions should also focus on the women at higher parity levels and those who are in advanced stages of pregnancy.

## Introduction

Worldwide, 42% (39.9–43.8%) of the pregnant women are anemic. In Africa, the estimated prevalence of anemia in pregnancy is 51.9–59.6% [1]. The occurrence of anemia shows substantial variation within and between populations [2]–[4].

The Ethiopian Demographic and Health Surveys (DHS) of 2005 and 2011 reported that 27% and 17% of Ethiopian women in the reproductive age were anemic, respectively [5], [6]. Similarly, other nationwide surveys of women in childbearing age documented a prevalence of 29.4% and 30.5% [7], [8]. Earlier studies among pregnant women conducted in different parts of Ethiopia indicate variation in prevalence of anemia that ranges 15–42% [9]–[12]. For instance, studies from urban settings of Awassa (southern), Jimma (southwestern), and Gondar (northwestern) reported 15%, 21.6% and 38.4% of anemia among pregnant women, respectively [10]–[12]. Moreover, a prevalence of 42% was documented by a community based study of pregnant women in Jimma town that indicates further variation of the existing body of knowledge [9].

Multiple factors cause anemia in pregnancy. The most common ones include iron deficiency [13], [14], deficiencies of other micronutrients such as vitamin A, folic acid, zinc, copper and other vitamins and minerals [15]–[18], frequent pregnancies [12], [14], and severe infections such as malaria and HIV/AIDS [19], [20]. Hookworm and other Intestinal helminthes infections [21], [22] and poor economic status also result in anemia [23].

Most of the previous studies on anemia in Ethiopia were conducted on samples of childbearing women, and few studies assessed anemia and its predictors among pregnant women in the country. As a result, there is little information about anemia during pregnancy in the rural communities of Ethiopia. Such data are needed to provide effective maternal health services, and to decrease maternal and neonatal morbidity and mortality, which are unacceptably high in Ethiopia. Therefore, this study was conducted to determine the prevalence of anemia and identify its predictors among pregnant women in the rural eastern Ethiopia.

## Materials and Methods

### Ethics

The protocol was reviewed and approved by Institutional Review Board of Haramaya University and the National Research Ethics Review Committee (NRERC) of Ethiopia. All women who took part in this study provided informed written consent. Married pregnant women below the age of 18 years were considered emancipated minors and as such allowed to provide independent consent. The inclusion of these emancipated minors in the study was approved by IRB, to provide independent informed consent.

### Study Design and Population

We conducted a community-based cross-sectional study in Haramaya district, eastern Ethiopia, from April to June 2010. The district is divided into 33 rural and 4 urban ‘kebeles”, which are the lowest units of administration in Ethiopia. The altitude of the district ranges from 1400 to 2340 meters above sea level and its estimated population is 236,600, of whom, more than 90% live in the rural kebeles. The livelihood of the people in the district is based on mixed farming with predominant crop production. Khat (*Catha edulis* Forsk) is the dominant cash crop widely produced in the area. According to the district health office, malaria is endemic in 4 of the rural kebeles. HIV/AIDS testing is routinely done for pregnant women in recent years to prevent mother to child transmission of the infection. Among the 2000 pregnant women who were screened for HIV/AIDS in 2010, three were positive.

### Sample Size Determination and Sampling Procedure

The minimum sample size required for this study was determined assuming a power of 80%, a confidence interval of 95%, a prevalence of 32% for anemia among women who do not consume vegetables [17] and a prevalence difference of 7.0% expected between the groups of women who consume vegetables frequently and those who do not. In addition, a 15% non-response rate was considered. Accordingly, the minimum sample size required for the study was 1600. Based on the available information about the average number of pregnant women in each kebele (cluster in this study), we estimated that the required sample size can be obtained from 21 of the 33 rural kebeles. Therefore, we obtained the list of the 33 rural kebeles and assigned a unique number to each of them in order to create a sample frame. Then, 21 kebeles were randomly selected from the sampling frame that consisted of 33 unique numbers, one for each kebele. We used a simple random sampling method (lottery technique) to select the kebeles (clusters).

In each selected kebele, a complete registration of pregnant women was performed. After registering women with known pregnancy in the selected kebeles, the rest were assessed for pregnancy using a screening checklist that consisted of six items with ‘Yes’ or ‘No’ response [24], [25]. The screening checklist asked about delivery and breast-feeding in the last 6 months, delivery in the last four weeks, menstrual period in the last seven days, abortion, or miscarriage in the last seven days, sexual abstinence since the last menstrual period and the current use of contraceptives. Then, the urine of the women who were suspected pregnant was tested for confirmation using a rapid test kit (human gesellschaft für biochemica and diagnostics mbh, Wiesbaden, Germany). A total of 1771 pregnant women were identified in the selected kebeles and all of them were recruited to increase the power of the study.

### Data Collection Methods and Measurement Procedures

Data were collected using three methods: interviewer-administered questionnaire, measurements of maternal anthropometry, and measurements of hemoglobin concentration. The questionnaire consisted of items on the respondents' socio demographic characteristics, dietary intake, feeding habits, health histories and some gynecological and obstetric variables. The study subjects' water source was considered as “protected” if it was from a pipe, a tanker or a well with hand pump, or “unprotected” otherwise. The gestational age of the women was estimated by reported Last Menstrual Period (LMP) and, for those who were unable to remember their LMP, by their report of the duration of their pregnancy. The items were adapted from the Ethiopian DHS 2005 and from a simplified Food Frequency Questionnaire (FFQ) as used in the country previously [26], [27].

The frequency of vegetable consumption was defined as once a week or more and this in turn was estimated from the responses to the FFQ as it was used in earlier Ethiopian studies [7], [8]. The cutoff point was chosen considering the frequency distribution of vegetable consumption in present study and the socio cultural norms of food consumption in the community.

The respondents' dietary intake was assessed by a single 24 hour dietary recall, and we used the recall to calculate their Dietary Diversity Scores (DDS) [27]. All the foods and the liquids consumed a day before the survey were categorized into 12 groups. Consuming a food item from any of the groups assigned a score of 1 and if no food was consumed a score of 0 was given. Accordingly, a DDS of 12 points was computed by combining the values of all the groups [27]. The DDS was categorized as low (< = 3), medium (4 or 5) and high (> = 6) [26].

Consuming iron rich foods was restricted to eating iron rich foods of animal source such as meat, organ meat, fish or poultry [26]. The women were also asked about a change made in their food intake habit after they had become pregnant. They were asked whether they increased or decreased the size or frequency of their meal after their pregnancy. The responses were aggregated to define restrictive dietary behavior. The study participants who reduced either the frequency or the size of their meal were identified as having restrictive dietary behavior while those who increased or maintained their meal size or frequency were identified as not having restrictive dietary behavior.

The anthropometric data were collected according to the WHO recommended standards [28]. Weight was measured by a digital scale; height was measured using a locally prepared wooden stadiometer, and left mid upper arm circumference (MUAC) was measured using insertion type non-elastic tape.

The women's hemoglobin concentration was measured by HemoCue® Hb 301 system, according to the manufacturer's instructions (HemoCue AB Ängelholm Sweden). A prick was made on the tip of the middle finger after the site was cleaned with disinfectant. The first two drops were wiped away and the third drop was used to fill the cuvettes for measuring hemoglobin concentration. The accuracy of this method has been established by previous studies [29] and this method is recommended for use in resource limited settings [30].

Anemia was defined as a hemoglobin concentration of <11.0 g/dl and hemoglobin levels of 10–10.9 g/dl, 7–9.9 g/dl and less than 7.0 g/dl were considered as mild, moderate and severe anemia, respectively [31]. Other cutoff points of hemoglobin concentration specific to trimesters of pregnancy are suggested by the International Nutritional Anemia Consultative Group (INACG) and others [32]. However, we used the same cutoff point regardless of the trimester of pregnancy. This was because all the earlier studies in Ethiopia and other recent publications have used the same cutoff points and the WHO has not yet recommended trimester specific cutoff points. To approximate the hemoglobin values at sea level, the measured values were adjusted using correction factors at every 500 meters for altitudes more than 1000 meters above sea level [32].

### Field Workers, Training, and Supervision

The data collectors were nurses and laboratory technicians; they were trained for the purposes of the study and were fluent speakers of the local language, Afan Oromo. Trained research assistants and the principal investigator supervised the data collection, provided onsite technical support for the data collectors, and collected and checked all the completed questionnaires daily. Training, field-testing, and standardization of measurements were carried out before the actual fieldwork. Morning meetings, led by the principal investigator, were held with all field workers throughout the data collection period and continuous feedbacks were provided.

### Statistical Analysis

Data were entered by two data entry clerks on different computers using Epidata Version 3.1 and analyzed using STATA Version 11.0 (College Station, TX USA). A multilevel mixed effect logistic regression model was used to adjust for the clustering effect. Kebele (cluster) of residence was considered as a random effect variable and other predictor variables were used as fixed effects. The STATA “xtmelogit” command was used to run the analysis [33].

The independent variables entered in the multivariable logistic regression model were grouped as socio-demographic information, gynecological/obstetric and health histories, and nutritional status and dietary habits of respondents. The respondents' socio-demographic variables were age, education, type of marital relationship and water source. The gynecological/obstetric and health histories were parity, trimester of pregnancy, history of malaria, use of iron/folic acid supplements, pregnancy intention and prenatal checkups, while nutritional status and dietary habits of respondents were dietary diversity level, consumption of iron rich foods, restrictive dietary behavior, vegetable consumption, khat chewing and MUAC. The conceptual model for the determinants of anemia for low and middle income countries was used to select the variables in multivariable logistic regression model [34].

Categorical variables were presented using percentages and frequency counts. Continuous variables were presented as mean and standard deviations. The association of the categorical variables with anemia was analyzed using chi-squared statistics. We used independent sample t-test and one way Analysis of Variance (ANOVA) to compare the mean hemoglobin levels among the categorical variables. Crude odds ratios (COR) and adjusted odds ratios (AOR) were used as measures of the associations in the bivariate and the multivariable analysis, respectively. The precisions of the measures of associations were given by their 95% confidence interval (CI). Deviance information criterion (DIC) was used to assess the adequacy of the adjusted model. Two-sided p-value of <0.05 indicates the statistical significance of the findings.

## Results

### Characteristics of Subjects and Prevalence of Anemia

A total of 1678 pregnant women participated in the study and included in the analysis. Thus, a response rate of 94.7% was achieved. Among the 93 pregnant women who were excluded from the analysis, 63 refused finger pricking for hemoglobin test and 30 gave birth before the data collection. There was no difference in the background characteristics between the women included in the analysis and those excluded from the analysis.

Almost all of the study participants were Oromo (99.6%) and Muslim (99.3%). Their mean age was 25.5 (SD±5.3) years and less than 10% of the study participants were older than 34 years. Most of the respondents had no formal education (92.0%); few were in polygamous marriage (13.6%), and nearly half (49%) were in the third trimester of pregnancy (Table 1). Little more than a quarter of the participants (27.9%) gave five or more births, whereas few gave no birth previously (18.7%). About one third of the respondents chewed khat daily (34.6%). In this study, 726 (43.3%) of the pregnant women had antenatal checkups, 151 (9.0%) received an iron/folic acid tablet supplementation during their pregnancy and 30 (1.8%) had reported history of malaria in 30 days prior to the study. The consumption of iron rich foods during the reference period was low and was reported only by 226 (13.5%) respondents. Anemia was identified in 737 (43.9%) study subjects (95% CI, 41.5%–46.3%). Among the anemic women, 47.6%, 46.8% and 5.6% had mild, moderate and severe anemia, respectively.

**Table 1:** Socio-demographic and some selected characteristics of pregnant women in Haramaya district, eastern Ethiopia 2010(n = 1678).

Variable	Frequency	Percent (%)
Age category of women		
15–19 years	153	9.1
20–24 years	475	28.3
25–29 years	552	32.9
30–34 years	372	22.2
35–49 years	126	7.5
Educational level		
No formal education	1546	92.1
Primary level	116	6.9
Secondary or higher	16	1.0
Polygamous marital relation		
Yes	233	13.9
No	1445	86.1
Livestock possession		
Yes	912	54.4
No	766	45.6
Agricultural land possessed		
No	598	35.6
Yes	1080	64.4
Gestational age		
First trimester	245	14.6
Second trimester	607	36.2
Third trimester	826	49.2
Parity level		
never gave birth	314	18.7
One birth	228	13.6
Two births	232	13.8
Three births	221	13.2
Four births	216	12.9
Five or more births	468	27.8
Khat chewing		
Less than daily	1097	65.4
Daily	581	34.6
Restrictive dietary habit		
No	1438	85.7
Yes	240	14.3

### Bivariate Analysis of Factors Associated with Anemia

In the bivariate analysis, the prevalence of anemia was significantly higher among the pregnant women who had restrictive dietary behavior than those who did not (p<0.05), and in women who chewed khat daily than those who sometimes or never did so (p<0.05). Similarly, anemia was higher in the second (p<0.001) and third (p<0.001) trimesters of pregnancy than in the first trimester. Higher proportions of anemic women were also observed among the respondents who gave 2 births (p<0.01), 3 births (p<0.05) and 4 or more births (p<0.01) than among those who did not give birth (Table 2).

**Table 2:** Predictors of anemia among pregnant women in Haramaya district, Eastern Ethiopia, 2010 (n = 1678): results of bivariate and multivariable logistic regression models.

Variable	Anemia status		COR (95% CI)	†AOR (95% CI)
	Not anemic freq. (%)	Anemic freq. (%)		
Marital type				
Polygamous	122 (52.4)	111 (47.6)	1.19 (0.90, 1.57)	1.24 (0.91, 1.69)
Monogamous	819 (56.7)	626 (43.3)	1	1
Educational level				
None	854 (55.2)	692 (44.8)	1	1
Primary	78 (67.2)	38 (32.8)	0.60 (0.40, 0.90)*	0.71 (0.45, 1.11)
Secondary +	9 (56.3)	7 (43.7)	0.96 (0.36, 2.59)	1.87 (0.64, 5.50)
Water supply				
unprotected	567 (52.5)	513 (47.5)	1.51 (1.23,1.85)***	1.20 (0.94, 1.53)
protected	374 (62.5)	224 (37.5)	1	1
Pregnancy intention				
Intended	683 (57.8)	498 (42.2)	1	1
Not intended	258 (51.9)	239 (48.1)	1.27 (1.03,1.57)*	1.29 (1.01, 1.65)*
Prenatal care				
No	562 (59.0)	390 (41.0)	1	1
Yes	379 (52.2)	347 (47.8)	1.32 (1.09, 1.60)**	1.16 (0.93, 1.45)
Pregnancy stage				
first	191 (78.0)	54 (22.0)	1	1
second	380 (62.6)	227 (37.4)	2.11 (1.50, 2.98)***	2.09 (1.46, 3.00)***
Third	370 (44.8)	456 (55.2)	4.36 (3.13, 6.07)***	4.23 (2.97, 6.02)***
Parity level				
Never gave birth	201 (64.0)	113 (36.0)	1	1
1 birth	144 (63.2)	84 (36.8)	1.04 (0.73, 1.48)	1.18 (0.80, 1.74)
2 births	120 (51.7)	112 (48.3)	1.66 (1.18, 2.35)**	1.68 (1.15, 2.47)**
3 births	117 (52.9)	104 (47.1)	1.58 (1.11, 2.24)*	1.60 (1.08,2.37)*
4 births +	359 (52.6)	324 (47.4)	1.61 (1.22, 2.11)**	1.41 (1.00, 2.00)
Dietary restriction				
No	826 (57.4)	612 (42.6)	1	1
Yes	115 (47.9)	125 (52.1)	1.47 (1.12, 1.93)**	1.39 (1.02, 1.88)*
Khat chewing daily				
No	649 (59.2)	448 (40.8)	1	1
Yes	292 (50.3)	289 (49.7)	1.43 (1.17, 1.76)***	1.29 (1.02, 1.62)*

The estimate for random effect  = 0.210 (0.094, 0.470) *** and Model fit statistics, -2loglikelihood (DIC) = 2079.5.

*p<0.05,

**p<0.01, and *** *p*<0.001: COR, crude odds ratio: CI, confidence interval: AOR, adjusted odds ratio: DIC, deviance information criterion:

† Adjusted for educational status, age, type of marriage, source of water supply, pregnancy intention, reported history of malaria, reported iron supplementation, antenatal care visit, trimester of pregnancy, number of previous births, mid upper arm circumference (MUAC), consumption of iron rich food in reference period, level of dietary diversity score, frequency of vegetable consumption, khat chewing daily and restrictive feeding behavior.

The mean hemoglobin level of the respondents was 11.0±1.7 g/dl. The women with a restricted dietary behavior had a mean hemoglobin level of 10.7±1.6 g/dl whereas this was 11.0±1.7 g/dl for those women without such behavior (t-test, p = 0.0001). There was no significant difference in mean hemoglobin level between low, medium and high DDS levels (ANOVA, p = 0.146). The risk of anemia was significantly associated with khat chewing among women not consuming iron rich food (OR, 1.45; 95% CI, 1.17–1.80) but not among those consuming iron rich food in the reference period (OR, 1.25; 95% CI, 0.70–2.26).

The risk of anemia was positively associated with the age of the women (COR, 1.03; 95% CI, 1.01–1.05), their history of malaria (COR, 2.24; 95% CI, 1.06–4.73) and their use of iron/folic acid supplements (COR, 1.58; 95% CI, 1.13–2.21). The risk of anemia was reduced by 29% among the women who consumed vegetables once or more in a week compared to those who did not (COR, 0.71; 95% CI, 0.57–0.88). Similarly, it was decreased as mid upper arm circumference of the women increased (COR, 0.94; 95% CI, 0.90–0.99).

There was no difference in the risk of anemia between the women who consumed iron rich foods (meat poultry or fish) in the reference period and those who did not (COR, 0.82; 95% CI, 0.62–1.10). Compared to the respondents with low DDS, the risk of being anemic was not significantly different for those with medium (COR, 0.80; 95% CI, 0.62–1.03) and high (COR, 0.86; 95% CI, 0.66–1.11) DDS levels.

### Predictors of Anemia in Multivariable Analysis

In the multivariable model, the respondents who chewed khat everyday had a 29% higher risk of anemia than those who did so occasionally or never (AOR, 1.29; 95% CI, 1.02–1.62). Moreover, the women with restrictive dietary behaviors were more likely to have anemia than those without (AOR, 1.39; 95% CI, 1.02–1.88). The risk of anemia was increased by 68% (AOR, 1.68; 95% CI, 1.15–2.47) and 60% (AOR, 1.60; 95% CI, 1.08–2.37) for women who gave 2 and 3 births compared to those who did not give birth before, respectively (Table 2). Compared to the study subjects who were in their first trimester, the women in the second and third trimesters had more than two-fold (AOR, 2.09; 95% CI, 1.46–3.00) and four-fold (AOR, 4.23; 95% CI, 2.97–6.02) higher risk of anemia, respectively (Table 2).

There was no significant association between the risk of being anemic and eating iron rich foods (AOR, 0.75; 95% CI, 0.50–1.14), history of malaria (AOR, 1.75; 95% CI, 0.75–4.09), and using iron/folic acid supplements (AOR, 1.09; 95% CI, 0.74–1.59). Furthermore, there was no statistically significant association of risk of anemia with source of water supply, maternal age, type of marriage and maternal education in a multivariable model (Table 2). However, the risk of anemia was negatively associated with MUAC (AOR, 0.92; 95% CI, 0.87–0.97) and consumption of vegetables (AOR, 0.75; 95% CI, 0.59–0.96).

## Discussion

Our results showed that anemia was highly prevalent among the pregnant women in the rural eastern Ethiopia. Chewing khat daily, restrictive dietary behavior including reduced meal size or frequency, advanced pregnancy stages and parity levels of 2 or more births were important predictors of anemia during pregnancy in the study population, which is relatively homogenous in terms of ethnicity, religion and other socio-cultural characteristics.

The prevalence observed in this study shows that anemia is a severe public health problem as classified by WHO [35]. Our observation is higher than the levels established for Ethiopian childbearing women [8], [26]. This difference may be attributed to the fact that the previous studies did not considered exclusive sample of pregnant women; they studied women of childbearing age. The level of anemia reported by present study is also higher than the findings of similar studies done in urban Ethiopia [10], [11]. However, our finding is almost similar to the 42% reported from the community based setting of south western Ethiopia [9]. It is important to note that direct comparison of our observation with earlier studies conducted in Ethiopia is impossible because of the differences in the number and composition of the study subjects enrolled, study settings and socio cultural circumstances.

The association of chewing khat frequently with higher risk of anemia in this study could be explained by the loss of appetite [36]. Furthermore, khat contains a substantial amount of tannin [37], which reduces the bioavailability of non-heme iron from the maternal diet that is mainly based on foods of plant sources in the population. The interaction between chewing khat and consuming iron rich foods in the stratified analysis for the risk of anemia further strengthened the possibility of our hypothesized inhibitory effect of khat on iron absorption.

An adequate nutrition during pregnancy is very useful to meet the increased demand of nutrients and prevent unwanted consequences [38]. Restrictive dietary behaviors during pregnancy result in inadequate food intake both in quality and in quantity. This may also lead to poor iron intake. The association between restrictive dietary habit and anemia in our study is consistent to the observation of the study conducted on pregnant women in Mali [39]. All these findings indicate the need for dietary counseling during pregnancy.

In addition, our study observed other important risk factors of anemia during pregnancy. The higher risk of anemia during second and third trimester of pregnancy observed in this study is similar to the findings of several previous studies [40]–[42]. These findings imply unmet needs for iron, which increase as the gestational age increases [43]. The association of the risk of anemia with increased parity of the women reported in this study is also demonstrated by previous works [44], [45]. The increased risk of anemia with the increased number of births can be explained by maternal depletion syndrome [44].

The findings of the present study suggest the importance of reducing the high level of anemia during pregnancy with the right intervention in the associated contextual factors. The women could be told about the negative effects of chewing khat frequently and be encouraged to increase their food intake during pregnancy. It is also possible to lower the risk of anemia by advising the women to consume iron rich foods and by providing iron/folic acid supplements, in addition to other existing prenatal health services. It has been demonstrated by previous work that prenatal counseling increases hemoglobin levels and effectively reduces the prevalence of anemia [46].

Further studies are needed to measure the effects of chewing khat on anemia among different population groups. Even though chewing khat is a fast spreading activity in Ethiopia and other parts of the world, including North America and Europe [47]–[50], its effect on health is not yet fully understood.

The reduced risk of anemia with frequent consumption of vegetables in the present study is in agreement with what has been demonstrated by Haidar and Pobocik [7]. This finding and the decreased risk of anemia with increased MUAC in our study indicate that encouraging frequent vegetable consumption and improving overall maternal nutrition may help in controlling the burden of anemia.

Contrary to the findings reported by Gebremedhin and Enquselassie [26], dietary diversity levels and consumption of iron rich foods did not affect the risk of anemia in present study. The lack of significant associations of the risk of anemia with consumption of iron rich foods and dietary diversity levels in our study should be taken with precaution. In present study, most of the respondents had low dietary diversity level and few reported consumption of iron rich foods, making the detection of variations difficult. Similarly, lack of significant association between the risk of anemia and maternal education in multivariable model could be due to the homogeneity of the respondents' educational status, as higher than 90% of respondents in present study had no any formal education.

Some limitations that might have affected the findings of present study should be noted. The first of these limitations is that the majority of the data were obtained from maternal self-reports. This may result in social desirability and recall biases. Nevertheless, chewing khat and restrictive feeding habits are not sensitive issues in the context; therefore, there is hardly any reason to doubt the responses obtained. In the context of population in our study area, chewing khat is an accepted social behavior and people are free to report it. However, the women may misreport their chewing habit as they were or were not chewing daily at the time of the interview that might or might not be consistent with their long-term khat chewing behavior. Additionally, the women who reported only a reduced meal frequency or only a reduced meal size might not necessarily have restrictive dietary behavior. This is because of the fact that a reduced meal frequency might be compensated by an increased meal size or a reduced meal size might be compensated by an increased meal frequency. However, the restrictive dietary behavior was obtained by aggregating the responses from multiple items that should have reduced such problems, since using multiple item scale is known to have a higher predictive validity in measurement [51].

The other limitation could be related to some unmeasured variables. Due to lack of resources, we did not measure the maternal status of some micronutrients as well as the presence of parasitic infections like helminthes and *Plasmodium falciparum*. Thus, it was not possible to assess or control for their potential effects. The deficiencies of these unmeasured micronutrients and the presence of the parasitic infections such as helminthes and malaria are seen as predictors of anemia. The unmeasured factors could also explain the higher level of anemia reported in this study, but these unmeasured factors might not have differential distribution between the categories of the predictors we identified.

Efforts were made at the planning and implementation phases of the study to ensure the quality of the data; the field workers were trained and the data collection was closely supervised. In addition, the hemoglobin values were measured at the end, after all the other data were obtained from the respondents. Therefore, any potential misreporting errors are unlikely due to maternal awareness of their hemoglobin level or anemia. Thus, it is likely that misreporting of exposure status could be non-differential. It is known that independent non-differential misclassifications of exposures cause bias of the risk estimates towards the null value [52]. Given the possibility of non-differential misreporting, the true effects of khat chewing and dietary restrictions on anemia during pregnancy in this study might have been underestimated.

## Conclusions

Overall, about two out of five pregnant women in our study suffered from anemia. Chewing khat everyday and restrictive dietary behaviors during pregnancy were associated with having anemia. Therefore, dietary counseling and reducing the habit of chewing khat during pregnancy are recommended. The counseling interventions should be integrated to community-based health services as components of the health extension program. The interventions should focus on improving the consumption of iron rich foods and on enhancing the provision of iron/folic acid supplements during pregnancy. Further studies are needed to evaluate the effects of chewing khat during pregnancy. In addition, the higher risk of anemia associated with higher parity levels and advanced stages of pregnancy implies the need of tailored interventions for these vulnerable groups of women.
